# Victors: a web-based knowledge base of virulence factors in human and animal pathogens

**DOI:** 10.1093/nar/gky999

**Published:** 2018-10-26

**Authors:** Samantha Sayers, Li Li, Edison Ong, Shunzhou Deng, Guanghua Fu, Yu Lin, Brian Yang, Shelley Zhang, Zhenzong Fa, Bin Zhao, Zuoshuang Xiang, Yongqing Li, Xing-Ming Zhao, Michal A Olszewski, Luonan Chen, Yongqun He

**Affiliations:** 1 Unit for Laboratory Animal Medicine, Department of Microbiology and Immunology, and Center for Computational Medicine and Bioinformatics, University of Michigan Medical School, Ann Arbor, MI 48109, USA; 2 Key Laboratory of Systems Biology, CAS Center for Excellence in Molecular Cell Science, Institute of Biochemistry and Cell Biology, Shanghai Institutes for Biological Sciences, Chinese Academy of Sciences, Shanghai 200031, China; 3 Department of Veterinary Medicine, Jiangxi Agricultural University, Nanchang, Jiangxi 330045, China; 4 Institute of Animal Husbandry and Veterinary Medicine, Fujian Academy of Agricultural Sciences, Fuzhou, Fujian 350013, China; 5 Division of Pulmonary and Critical Care Medicine, Department of Internal Medicine, University of Michigan Health System and Research Service, VA Ann Arbor Health Systems, Ann Arbor 48109, MI, USA; 6 Institute of Animal Husbandry and Veterinary Medicine, Beijing Municipal Academy of Agriculture and Forestry Sciences, Beijing 100097, China; 7 Institute of Science and Technology for Brain-Inspired Intelligence, Fudan University, Shanghai 200433, China; 8 CAS Center for Excellence in Animal Evolution and Genetics, Chinese Academy of Sciences, Kunming, Yunnan 650223, China; 9 School of Life Science and Technology, Shanghai Tech University, Shanghai 201210, China

## Abstract

Virulence factors (VFs) are molecules that allow microbial pathogens to overcome host defense mechanisms and cause disease in a host. It is critical to study VFs for better understanding microbial pathogenesis and host defense mechanisms. Victors (http://www.phidias.us/victors) is a novel, manually curated, web-based integrative knowledge base and analysis resource for VFs of pathogens that cause infectious diseases in human and animals. Currently, Victors contains 5296 VFs obtained via manual annotation from peer-reviewed publications, with 4648, 179, 105 and 364 VFs originating from 51 bacterial, 54 viral, 13 parasitic and 8 fungal species, respectively. Our data analysis identified many VF-specific patterns. Within the global VF pool, cytoplasmic proteins were more common, while adhesins were less common compared to findings on protective vaccine antigens. Many VFs showed homology with host proteins and the human proteins interacting with VFs represented the hubs of human–pathogen interactions. All Victors data are queriable with a user-friendly web interface. The VFs can also be searched by a customized BLAST sequence similarity searching program. These VFs and their interactions with the host are represented in a machine-readable Ontology of Host–Pathogen Interactions. Victors supports the ‘One Health’ research as a vital source of VFs in human and animal pathogens.

## INTRODUCTION

Infectious diseases caused by pathogenic microorganisms remain globally a major source of human mortality (1), and veterinary infectious diseases result in significant losses of livestock, pets and wildlife (2). Domestic animals and wild primates are also major sources of human infectious diseases (3). Approximately 60% of all infectious diseases in humans are zoonotic diseases that are transmissible from animals to humans (4); however, there is still a big gap in understanding the underlying mechanism. To more effectively study the zoonotic diseases and achieve better public health outcomes, the emerging ‘One Health’ movement aims to unite human and veterinary medicine and integrate human, animal and environmental health (5). Understanding the molecular mechanisms that enable pathogens to cause infectious diseases is critical to find effective treatment strategies for combating human and animal infectious diseases. In particular, further studies of virulence factors (VFs) that allow microbial pathogens to evade host defense mechanisms and cause disease will improve rational vaccine and drug design for preventing and treating infectious diseases.

Currently, several VF databases are available online. Among them, the Virulence Factors of Pathogenic Bacteria (VFDB) is a database of bacterial VFs with a focus on human bacterial pathogens (http://www.mgc.ac.cn/VFs/) (6). VFDB does not include VFs from parasites, fungi or viruses. VFDB only provides general VF references without the detail of the experimental verification evidences. Another database, PHI-base (http://www.phi-base.org/) contains genes involved in host–pathogen interactions. In addition to its primary focus on plant pathogens, PHI-base also contains medically important pathogens that can cause human diseases, and the database provides links to appropriate publications (7). In addition to VFs, PHI-base also includes pathogen proteins that interact with host without known effect on pathogenesis (7). The PATRIC bacterial bioinformatics database BLASTs all genomes, both public and private, against a database of virulence factors and displays those genes with homology on a page showing information about the genome and on a page for specialty genes of interest (8,9). PATRIC imports the VFs from VFDB and Victors (the database introduced in this paper). In addition, PATRIC has manually curated virulence factors for some bacterial pathogens and provides integrative data annotation and analysis (10).

To address the need for a comprehensive, curated database of human, animal and zoonotic pathogen VFs and to improve our understanding of the complexities of host–pathogen interactions, the Victors database was created. Victors is a manually curated, web-based (http://www.phidias.us/victors) VF database and analysis engine for human and animal pathogens. As an independent program in the PHIDIAS pathogen–host interaction database (11), whose original focus was on integrating existing sequence and disease information from various human and animal pathogens, the Victors VF database has an increased emphasis on the manual annotation and analysis of VFs. Each of the VFs in Victors is associated with manually annotated evidence from at least one peer-reviewed citation. It currently has over 5000 VFs from over 100 bacterial, viral, parasitic and fungal pathogens. The characteristics and functions of these VFs were extracted or analyzed using bioinformatics tools. Furthermore, Victors enabled us to make novel predictions about the interactions between specific proteins of the human host and experimentally verified microbial VFs.

## SYSTEM DESIGN, ANNOTATION, ANALYSIS PIPELINE AND STATISTICS

### System and database design

Victors is implemented using a three-tier architecture built on two University of Michigan virtual servers that run the Redhat Linux operating system (Redhat Enterprise Linux ES 4). Users can submit database or analysis queries through the web. These queries are then processed using PHP/SQL (middle-tier, application server based on Apache) against a MySQL (version 5.0) relational database (back-end, database server). The result of each query is then presented to the user in the web browser. Two servers are scheduled to back up data every week. Victors is a relatively independent program of the PHIDIAS database and analysis resource (11).

### Semi-automatic annotation of VFs

The semi-automatic Victors annotation system was developed by modifying an in-house web-based literature mining and curation system called Limix (11) (Supplementary Figure S1). The interactive Limix data submission and review system: (i) allows a curator to search literature, copy and edit text, and submit data to the database and (ii) provides a data reviewer tools to review, edit and approve the curated data on one comprehensive web interface. The major criterion to classify a VF is the loss or reduction of pathogenicity in the host after the VF gene mutation. Limix also features automated reference tracking and management. Upon approval after critical review by a domain expert (i.e. a scientist who is knowledgeable in the domain and has at least a postdoctoral position), the data will be posted publicly.

### VF Statistics

Victors currently contains 5296 VFs from 126 pathogens. Specifically, Victors includes 1160 VFs from 15 Gram-positive pathogens, 3488 VFs from 36 Gram-negative pathogens, 179 VFs from 54 viruses, 105 VFs from 13 parasitic pathogens and 364 VFs from 8 fungal pathogens that primarily infect humans and other animal species (Table 1 and Supplementary Table S1 shows representative statistics). More detailed statistical information is available at http://www.phidias.us/victors/stats.php.

**Table 1.** Representative Victors statistics as of 14 August 2018

#	Pathogen	No. of VFs	No. of human PPIs
**G+ Bacteria: out of 1160 VFs from 15 pathogens**			
1	*Bacillus anthracis*	54	–
2	*Clostridium botulinum*	7	470-2*
3	*Listeria monocytogenes*	106	–
4	*Mycobacterium tuberculosis*	360	192-11
5	*Streptococcus agalactiae*	30	1,224-12
6	*Streptococcus pneumoniae*	413	1549-18
7	*Streptococcus pyogenes*	77	2144-12
**G- Bacteria: out of 3488VFs from 36 pathogens**			
1	*Brucella* spp.	439	2203-221
2	*Escherichia coli*	569	1412-84
3	*Haemophilus influenzae*	52	9-4
4	*Legionella pneumophila*	68	48-9
5	*Neisseria meningitidis*	175	1507-21
6	*Salmonella* spp.	387	1765-16
7	*Shigella* spp.	349	2024-84
**Viruses: out of 179 from 54 pathogens**			
1	Feline infectious peritonitis virus	5	–
2	Herpes simplex virus type 1 and 2	9	–
3	Pseudorabies virus	9	–
**Parasites: out of 105 from xx pathogens**			
1	*Plasmodium* spp.	11	–
2	*Toxoplasma gondii*	9	–
**Fungi: out of 232 from xx pathogens**			
1	*Candida albicans*	108	216-12
2	*Cryptococcus neoformans*	66	–

**Notes:** 470-2*: represents 470 human proteins interacting with 2 virulence factors. This table includes pathogens with at least five virulence factors collected in Victors. More detailed information is available in Supplementary Tables S1 and S2.

### Bioinformatic analyses

The Clusters of Orthologous Group (COG) categories of VFs were retrieved from the NCBI COG database (12). The protein MW and PI are calculated from the protein sequences using the Bioperl program (https://bioperl.org/) (13). The Vaxign analysis pipeline (14) was used to analyze the subcellular localization, adhesin probability and conserved domains of VFs. Customized BLAST was used for BLAST sequence similarity search (15). For the prediction of host–pathogen protein–protein interactions (PPIs), the orthologs of proteins were defined by employing Inparanoid program (http://inparanoid.cgb.ki.se/) with default parameters (16), and the PPI database HPRD (http://www.hprd.org/) was used to identify the PPIs (17). More detail about PPI prediction is provided in Supplementary Figure S2.

### Database contents

**Figure 1.**
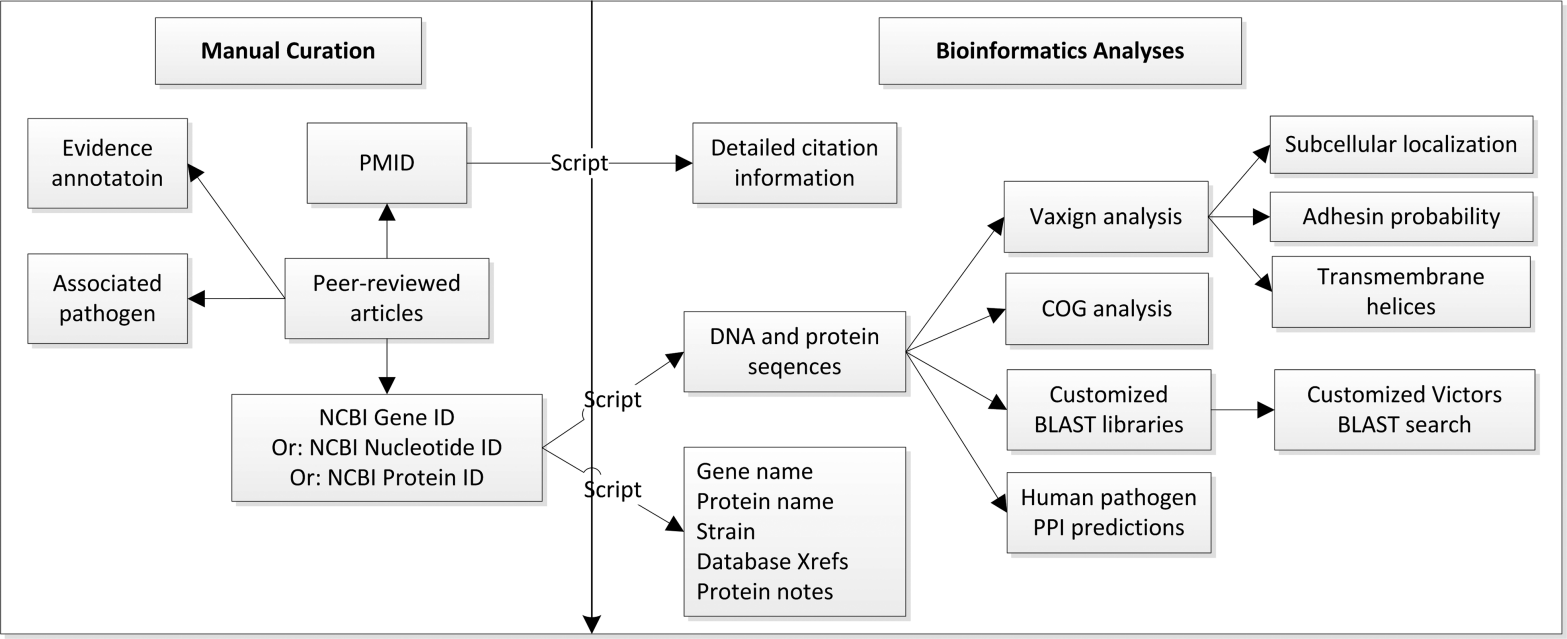
The workflow of the Victors virulence factor annotation and analysis. .

For each specific VF, the Victors database contains the following information: (i) general information on each VF gene symbol, protein name, general gene/protein functions, COG category (if available), and DNA and protein sequences; (ii) manually annotated text from peer-reviewed article that proves the status of VF and (iii) computationally calculated results for each VF, for example, protein weight, protein pI, subcellular localization, adhesin probability, conserved domains of VFs and predicted PPIs (Figure 1). Supplementary Table S2 summarized the COG functional classification of all Victors VFs and *Brucella* VFs stored in Victors. More analysis results are described below.

## VF DISTINCT PATTERNS COMPARED TO PROTECTIVE ANTIGENS

Previous studies found that protective antigens used for successful vaccine development are typically enriched in the areas of adhesin proteins, extracellular or cell surface bacterial proteins, and do not have sequence similarity to host proteins (18–20). We first compared the proportions of adhesin proteins in VFs and the vaccine immunogen proteins. We found that only 4% of viral VFs are adhesins or adhesin-like proteins (Table 2), while in the remaining types of pathogens adhesins or adhesin-like proteins represent 9–13% of VFs. Using the same Vaxign analysis method (14), it was previously found that over 50% of protective antigens used for development of experimentally verified vaccines are adhesins or adhesin-like proteins (20,21), showing that the VFs and protective antigens exhibit principally distinct molecular patterns.

**Table 2.** Vaxign analysis of Victors virulence factors

Pathogens	G+ bacterium	G- bacterium	Viruses	Parasites	Fungi	Total
Total VFs analyzed	1160	3489	179	105	364	5297
Predicted adhesin	124 (0.11)	439 (0.13)	8 (0.04)	9 (0.09)	19 (0.05)	599 (0.11)
Similarity to human proteins	292 (0.25)	665 (0.19)	21 (0.12)	30 (0.29)	180 (0.49)	1188 (0.22)
Similarity to mouse protein	293 (0.25)	670 (0.19)	21 (0.12)	31 (0.3)	181 (0.5)	1196 (0.23)
Extracellular	117(0.1)	274 (0.08)	–	–	–	–
Cell wall	73 (0.06)	–	–	–	–	–
Outer membrane	–	282 (0.08)	–	–	–	–
Cytoplasmic	792 (0.68)	2021 (0.58)	–	–	–	–
Cytoplasmic membrane	282 (0.24)	560 (0.17)	–	–	–	–
Periplasmic	–	165 (0.05)	–	–	–	–
Unknown	152 (0.13)	543 (0.16)	–	–	–	–

We further examined the frequency of VFs that possess human orthologs, which were detected as defined in reference (14), among different groups of pathogens. Out of 4749 proteins, 1188 (22%) are homologous to some human proteins (Table 2). Among them, 30 (29%) out of 109 parasitic proteins and 180 (49%) out of 364 fungal proteins have orthologs in human proteins. In contrast, only 21 (12%) out of 179 viral proteins have human orthologs. There are 665 (19%) and 292 (25%) VFs from Gram-negative and Gram-positive bacteria, respectively, which have homology to human proteins. The results suggest that fungi and parasites, both eukaryotic organisms, could much more frequently use the ‘orthologue strategy’ to interact with the host and as a mechanism of virulence. Similar results were obtained when the VFs were compared with the mouse proteome (Table 2).

Finally, we examined the subcellular locations of VFs among different groups of pathogens, including distinctions between Gram+ and Gram- bacterial VFs. Table 2 also shows the statistics of the cellular locations of virulence factors in Gram+ and Gram- bacterial VFs. Interestingly, 82% and 75% of Gram+ and Gram- bacterial VFs are located in the cytoplasmic and cytoplasmic membrane areas. However, the portions of extracellular or cell wall (or outer membrane) proteins are relatively low, representing <20% of the total bacterial VFs. These results indicate that key bacterial VFs are ‘designed’ to work inside the bacterial cells rather than on the surface or as secreted molecules. These results contrast with the patterns of bacterial protective antigens, where over 50% protective antigens are located on the cell surface or secreted out (20). Therefore, similar to the adhesin and ortholog studies reported above, the VFs and protective antigens also follow differential patterns of subcellular localization.

## PREDICTION OF HOST–PATHOGEN INTERACTIONS BASED ON VICTORS

**Figure 2.**
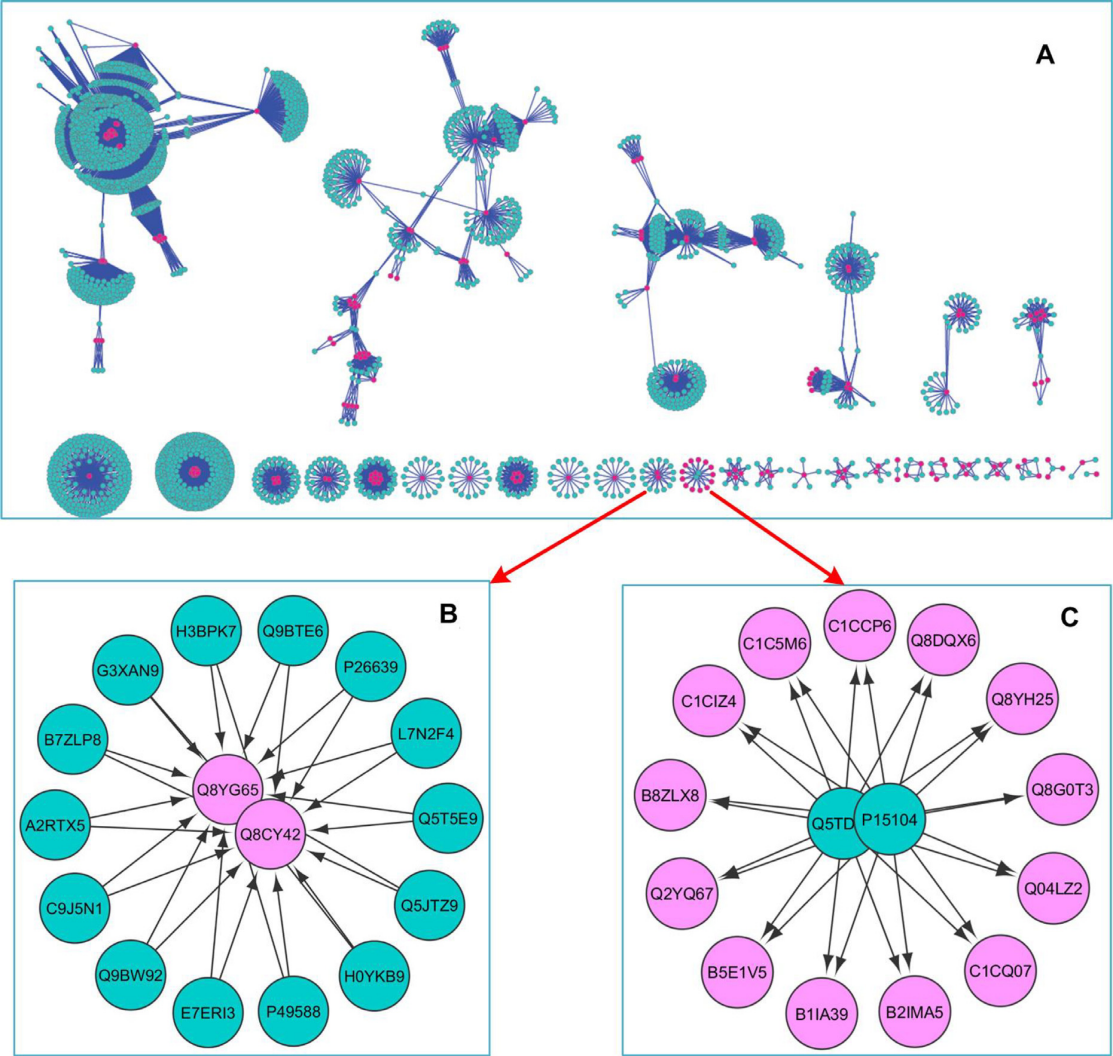
Landscapes of predicted bacteria pathogen–human PPIs. (**A**) All 15 334 PPIs. (**B**) This figure shows that two pathogen proteins (GPT pyrophosphokinase rsh) from *Brucella suis* (Q8CY42) and *Brucella melitensis* (Q8YG65) interact with a group of human proteins (blue dots) on tRNA level, such as t-RNA editing proteins, tRNA ligase proteins. The GO enrichment analysis reveals that this group of proteins is enriched in tRNA aminoacylation (A2RTX5|Q9BW92|Q5JTZ9|P49588|P26639), corrected *P*-value 9.5487E-12. (**C**) This figure shows that two human proteins lengsin (Q5TDP6) and Glutamine synthetase (P15104) interact with a group of pathogen proteins from different species, such as *Streptococcus pneumonia* and *Brucella suis, Brucella melitensis*. Interestingly, all pathogen proteins are Glutamine synthetase. Nodes in red color are pathogen proteins, and nodes in blue are human proteins.

Our next goal was to assess the value of Victors in predicting pathogen–human PPIs based on the analyses of protein domains and phylogeny. Using all of the VFs experimentally verified in the human system, we searched for potential interactions among the host proteins. As detailed in Supplementary Figure S2, candidate PPIs were generated using ‘domain inferred’ and ‘ortholog inferred’ predictions (22–24). The ‘domain inferred prediction’ was used to infer inter-species PPIs based on the domain–domain interactions (DDI). Such DDI inference is theoretically supported by the conservation and importance of domains in proteins (24). If one domain in the pathogen protein A interacted with another domain in the human protein B (domain interacts with domain), we inferred these two proteins could potentially interact (protein A interacts with protein B). The ‘ortholog inferred prediction’ infers the inter-species PPIs based on their orthologs. If a specific protein A interacts with another protein B and has an ortholog protein C, we inferred that protein B and C interact with each other. The interactions generated using ‘domain inferred’ and ‘ortholog inferred’ methods were then combined into a unique human–pathogen interactome (Figure 2 and Supplementary Figure S2).

Our initial candidate PPI predictions based on the ortholog and domain interfaces used all the proteins from individual pathogens. However, the results included many false positives since some pathogen proteins may not interact with host proteins due to various reasons (e.g. physical barriers). In order to increase the quality of the PPI predictions, we utilized experimentally verified VFs in Victors to screen candidate pairs. Based on our literature annotation, 553 VFs from 30 pathogen species were confirmed experimentally using human-specific analysis approaches (e.g. human cells). The PPI pairs that had no Victors’ VFs were removed. Finally, we inferred 15 334 human–pathogen interactions comprised of 185 VFs from 19 pathogen species and 2502 human proteins (Table 1 and Supplementary Table S3).

Bacterial VFs interact with host (e.g. human) proteins in the complex process of bacterium–host interactions. Our approach predicted 15 334 bacteria–human PPIs from 11 bacteria (Supplementary Figure S3). For each of the 15 334 PPIs, we calculated the degree of proteins and then plotted the density curves (Supplementary Figure S3), which uncovered higher degree of VFs interactions with human proteins, compared to that of the entire bacterial proteome. These findings point out that relative to the non-VF bacterial proteins, VFs are much more likely to interact with human proteins.

To visualize the landscape of predicted bacteria–human PPIs, Cytoscape (25) was utilized to display the interaction networks and perform GO enrichment analyses (Figure 2). The results revealed that most of the interactions were well-connected and formed 31 component networks in 7 specific network patterns (Figure 2). Interestingly, many proteins are found at the nexus of ‘linkages’ or ‘hubs’ of large numbers of interactions. To further explore these multiple targeted proteins, we calculated the number of interacting bacterial VFs for each human protein (Supplementary Table S3). For example, each of two cell adhesion functional proteins, P13611 (Versican core protein) and Q96GW7 (Brevican core protein), connects to 17 VFs, consistent with the process of attaching to human cells being required for bacterial virulence.

## DATABASE QUERY AND ACCESS

### Database query and Blast analysis

**Figure 3.**
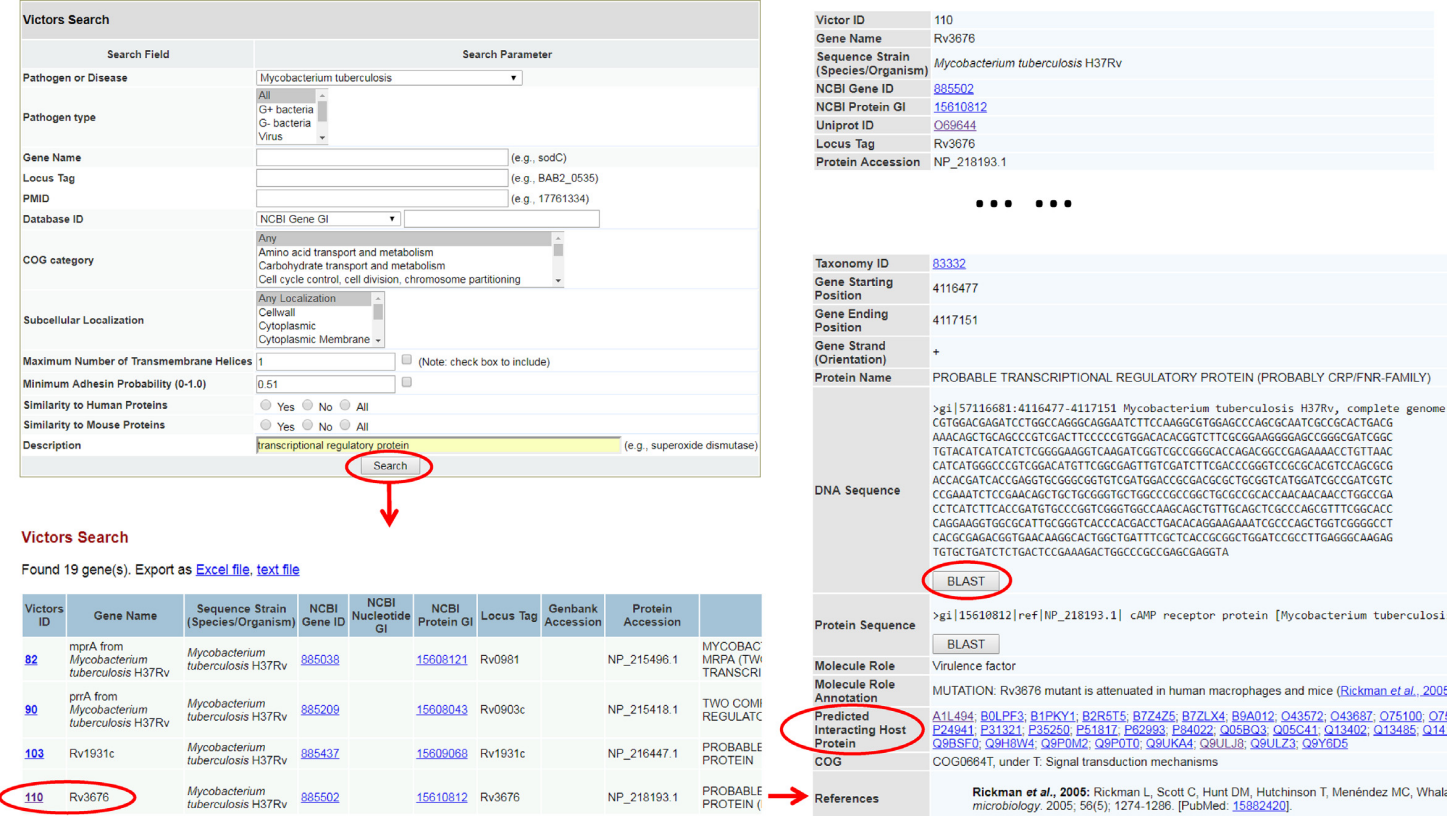
Example of Victors data query and BLAST sequence similarity analysis. In this example, a user selected ‘*Mycobacterium tuberculosis*’ in the ‘Pathogen or Disease’ menu bar and typed ‘transcriptional regulatory protein’. After clicking the ‘Search’ button, a list of 19 genes was shown up in the results page. A click on the Victors ID ‘110’ (the forth on the list) led to another page providing details about the VF (e.g. the gene mutation phenotype information and predicted interacting host proteins). A BLAST search was initiated by clicking ‘BLAST’.

The manually curated and pre-computed VFs data can be efficiently queried specifying one or multiple criteria: (i) gene or protein name, (ii) pathogen species or strain, (iii) locus tag of a VF, (iv) other existing database identifiers, such as NCBI Gene ID, NCBI nucleotide or protein GI, (v) keywords search with Boolean mode support, (vi) COG category, (vii) subcellular localization, (viii) four limiting factors, including the maximum number of transmembrane helices, the minimum adhesin probability and protein sequence similarity to human proteins, and protein sequence similarity to mouse proteins. User-friendly web interfaces are provided to display the results (Figure 3).

To facilitate antigen comparison and dynamical sequence analysis, two customized BLAST libraries have been generated. One library contains the protein sequences of all VFs in Victors, and the other contains all of the DNA sequences from all VFs. Victors users are able to perform BLAST sequence similarity searches using different BLAST tools.

### Data transfer and download

Several methods are provided in Victors to facilitate data exchange and transfer. First, a Victors web page (http://www.phidias.us/victors/download.php) is available for users to freely download the DNA and protein sequences of all VFs. In addition, after a web query from the Victors query website, a user can export queried VF information into a Microsoft Excel document.

We have also created an Ontology of Host–Pathogen Interactions (OHPI; https://github.com/OHPI/ohpi/). OHPI is developed by following the Open Biomedical Ontology (OBO) Foundry principles (e.g. openness and collaboration) (26). OHPI represents the VFs and how the mutants of VFs in Victors become less virulence inside a host organism or host cells. Currently, OHPI includes over 6700 terms. OHPI reuses existing ontologies (27). For example, OHPI reuses terms from the Gene Ontology (GO) (28), Ontology of Genes and Genomes (OGG) (29) and Cell Line Ontology (CLO) (30) to represent cellular components, genes and cell lines, respectively. OHPI includes object properties to semantically represent the relations between VFs and host entities (Supplementary Figure S4A). For example, the OHPI object property ‘gene mutant attenuated in host cell’ represents a relation between a gene and a host cell where the microbial mutant lacking the gene is attenuated in the host cell compared to the wild-type microbe. Such an object property can be used to represent a VF and its interaction in a host cell, for example, an oxidative stress response gene *ahpC* of *M. tuberculosis* strain H37Rv and mouse macrophage cell line J774 cell line (Supplementary Figure S4B), where the *ahpC* mutant of strain H37Rv is attenuated in J774 cells (31). Using the machine-readable Web Ontology Language (OWL) format (http://www.w3.org/TR/owl2-quick-reference), OWL-based software programs can be developed to parse and extract the information from the ontology and offer advanced data analysis. For example, using an OHPI SPARQL program (http://www.phidias.us/ohpi/sparql/), we could generate a simple SPARQL query script to identify 386 VFs whose mutants are attenuated in macrophages (Supplementary Figure S5A) and extract detailed information about the gene names, OGG IDs and VF annotations of these VFs (Supplementary Figure S5B).

### Availability and requirements

Victors is freely available for usage on the website http://www.phidias.us/victors. The Victors system can be accessed using a web browser.

## DISCUSSION

This article is the first formal introduction of the Victors database, a comprehensive knowledge base focused on experimentally verified VFs for human and veterinary pathogens. In addition to all the manually curated VFs data, Victors also provides the OHPI ontology representation and computational analysis of the data and user-friendly web interfaces for querying and analyzing the results. Its usage will support researchers in the areas of microbiology, vaccinology and immunology with curated data and bioinformatics tools.

Victors differs from existing VF databases in several significant ways. In addition to bacterial VFs as shown in VFDB (6), Victors includes VFs from viruses, parasites and fungi. While VFDB lists general references for a set of genes, Victors provides references for individual VFs and records the details on the experimental verification. Compared to PHI-base, which focuses on plant pathogens (7), Victors focuses on human and veterinary pathogens. The system design and data types for each VF among Victors, VFDB and PHI-base are also different. The Victors VF records are also semantically represented in the OHPI ontology. Data in the Victors database have been incorporated for many years by the PATRIC Specialty Genes resource (http://patricbrc.org/portal/portal/patric/SpecialtyGenes), a well-recognized bacterial bioinformatics database and analysis resource (9,10). While PATRIC can display Victors data, PATRIC does not provide all the details for each VF and is not responsible for the original annotation.

The predicted interactions between human proteins and VFs provided by Victors have improved our understanding of the pathogenesis of these pathogens. We found that among all pathogen proteins, VFs have higher tendency to interact with human proteins (Supplementary Figure S3). There are two possible explanations for this phenomenon. First, pathogens tend to use the VFs to hijack host defense pathways for the benefit of microbial pathogenesis. Second, human proteins tend to interact with the VFs in order to fight against the pathogen virulence mechanism. Using human–pathogen PPIs, Dyer *et al.* demonstrated that pathogens preferentially interact with human proteins that are hubs and bottlenecks in human interaction pathways (32,33). However, these studies by Dyer *et al.* do not differentiate pathogen VFs with other pathogen proteins that are not VFs and thus do not typically significantly contribute to microbial pathogenesis to a significant degree. In contrast, our study provides the evidence that compared to other pathogen proteins, pathogen VFs are more likely to interact with human proteins. Moreover, the human–pathogen interactomes can be used to identify which biological pathways are possibly utilized by the pathogens to invade the host and to develop candidates for future experimental validation.

We intend to keep adding new information to the resource. Our semi-automatic annotation pipeline makes the annotation process efficient. The manual curation and verification process (Supplementary Figure S1) ensures that every VF collected on the website has solid experimental evidence. Meanwhile, we are also testing new literature mining methods to ensure faster VF identification from literature. We also plan to mine and add more experimental conditions used for VF identification in the reported publications. As another future work, in contrast to the correlations between various factors, we will further add their direct associations or causal relations (i.e. network) to Victors by data-driven network methods (34).

Victors is expected to become a central and vital source of VFs and their associated data and support the One Health initiative. It is estimated that ∼75 percent of emerging human pathogens in the past 25 years have originated in animals, and the risk of zoonotic transmission is likely to increase in the future (35). In recent years, the One Health concept has gained well recognition in the public health and animal health communities. Its usage will support researchers in the areas of microbiology, vaccinology and immunology with curated data and bioinformatics tools.

## Supplementary Material

Supplementary Data
