# Supplementary material for: Victors: a web-based knowledge base of virulence factors in human and animal pathogens
Source: Nucleic Acids Res. 2018 Oct 26;47(Database issue):D693–700. doi: 10.1093/nar/gky999 (PMC6324020; doi:10.1093/nar/gky999)
Supplement: Supplementary Data [file gky999_supplemental_files.pdf]

Supplemental Materials of

Victors: a web-based knowledge base of virulence factors in human and animal pathogens

Samantha Sayers[1*], Li Li[2*], Edison Ong[1], Shunzhou Deng[1,3], Guanghua Fu[1,4], Yu Lin[1], Brian Yang[1], Shelley Zhang[1], Zhenzong Fa[1], Bin Zhao[1], Zuoshuang Xiang[1], Yongqing Li[5], Xing-Ming Zhao[6], Michal A. Olszewski[1,7], Luonan Chen[2,8,9,ξ], Yongqun He[1,ξ]

1. University of Michigan Medical School, Ann Arbor, MI 48109, USA
2. Key Laboratory of Systems Biology, CAS Center for Excellence in Molecular Cell Science, Institute of Biochemistry and Cell Biology, Shanghai Institutes for Biological Sciences, Chinese Academy of Sciences, Shanghai 200031, China
3. Department of Veterinary Medicine, Jiangxi Agricultural University, Nanchang, Jiangxi 330045, China
4. Institute of Animal Husbandry and Veterinary Medicine, Fujian Academy of Agricultural Sciences, Fuzhou, Fujian, China.
5. Institute of Animal Husbandry and Veterinary Medicine, Beijing Municipal Academy of Agriculture and Forestry Sciences, Beijing 100097, China.
6. Institute of Science and Technology for Brain-Inspired Intelligence, Fudan University, Shanghai, China.
7. VA Ann Arbor Healthcare System Research Service (11R), Ann Arbor, MI, USA
8. CAS Center for Excellence in Animal Evolution and Genetics, Chinese Academy of Sciences, Kunming 650223, China
9. School of Life Science and Technology, ShanghaiTech University, Shanghai 201210, China

* Co-first authors

ξ Co-corresponding authors

Supplemental Tables:

Supplemental Table 1. Representative Victors statistics as of August 10, 2018.
Supplemental Table 2. Summary of COG functional classification of Virulence factors in Victors.
Supplemental Table 3. Predicted PPI interactions between human proteins and virulence factors in Victors.

Supplemental Figures:

Supplemental Figure 1. Victors data curation, submission, and review processes.
Supplemental Figure 2. Project workflow for predicting pathogen-human protein-protein interactions.
Supplemental Figure 3. The density curves of protein degrees.
Supplemental Figure 4. OHPI ontology design pattern.
Supplemental Figure 5. OHPI SPARQL example.

Supplemental Table 1. Representative Victors statistics as of August 14, 2018.

Index	Pathogen Name	Disease	No. of VFs
		**G+ Bacteria (out of 1160)**	
1	*Bacillus anthracis*	Anthrax	54
2	*Clostridium botulinum*	Botulism	7
3	*Clostridium perfringens*	Food poisioning, Gas gangrene	10
4	*Enterococcus spp.*	bacteremia, endocarditis, and meningitis	42
5	*Listeria monocytogenes*	Listeriosis	106
6	*Mycobacterium tuberculosis*	Tuberculosis	360
7	*Staphylococcus aureus*	Staph infections	47
8	*Streptococcus agalactiae*	Group B streptococcus (GBS) infection	30
9	*Streptococcus pneumoniae*	Pneumonia	413
10	*Streptococcus pyogenes*	Strep throat, Scarlet fever	77
		**G- Bacteria (out of 3488)**	
1	*Actinobacillus pleuropneumoniae*	porcine pleuropneumonia	122
2	*Aeromonas salmonicida*	Furunculosis, Bald sea urchin disease	9
3	*Bartonella spp.*	Cat-scratch disease, endocarditis, trench fever	14
4	*Bordetella avium*	Bordetellosis	6
5	*Bordetella bronchiseptica*	Infectious bronchitis, kennel cough	5
6	*Bordetella pertussis*	Whooping Cough	24
7	*Borrelia burgdorferi*	Lyme Disease	18
8	*Brucella spp.*	Brucellosis	439
9	*Burkholderia mallei*	Glanders	10
10	*Burkholderia pseudomallei*	Melioidosis	61
11	*Campylobacter jejuni*	Campylobacterosis	81
12	*Escherichia coli*	Hemorrhagic colitis	569
13	*Francisella tularensis*	Tularemia	316
14	*Haemophilus influenzae*	Meningitis	52
15	*Helicobacter pylori*	Ulcers	90
16	*Legionella pneumophila*	Legionnaires' disease, Pontiac Fever	70
17	*Neisseria meningitidis*	Meningitis	175
18	*Pasteurella multocida*	Pasteurellosis, fowl cholera	36
19	*Pseudomonas aeruginosa*	Pseudomonas aeruginosa infection	105
20	*Salmonella* spp.	Salmonellosis	387
21	*Shigella*	Shigellosis	349
22	*Vibrio cholerae*	Cholera	199
23	*Yersinia enterocolitica*		59
24	*Yersinia pestis*	Plague	238
25	*Yersinia pseudotuberculosis*		33
		**Viruses (out of 179)**	
1	Feline infectious peritonitis virus	Feline infectious peritonitis (FIP)	6
2	Herpes simplex virus type 1 and 2	Herpes	22
3	Pseudorabies virus	Aujeszky's disease	12
		**Parasites (out of 105)**	
1	*Plasmodium* spp.	Malaria	21
2	*Toxoplasma gondii*	Toxoplasmosis	25
		**Fungi (364)**	
1	*Aspergillus fumigatus*		33
2	*Candida albicans*	Thrush, Candidiasis, yeast infection	120
3	*Candida glabrata*	Tortulosis	9
4	*Cryptococcus neoformans*		186
5	*Saccharomyces cerevisiae*		6

Note: This table includes pathogens with at least five virulence factors collected in Victors.

Supplemental Table 2: Summary of COG functional classification of Virulence factors in Victors and the COG classification for *Brucella* VFs.

COG Category	# of VFs in Victors	# of *Brucella* VFs
Amino acid transport and metabolism	375	76
Carbohydrate transport and metabolism	231	51
Cell cycle control, cell division, chromosome partitioning	27	0
Cell motility	185	16
Cell wall/membrane/envelope biogenesis	365	30
Chromatin structure and dynamics	1	0
Coenzyme transport and metabolism	123	12
Cytoskeleton	0	0
Defense mechanisms	51	4
Energy production and conversion	131	25
Extracellullar structures	0	0
Function unkown	172	4
General function prediction only	220	19
Inorganic ion transport and metabolism	246	21
Intracellular trafficking, secretion, and vesicular transport	259	20
Lipid transport and metabolism	89	10
Nuclear Structure	0	0
Nucleotide transport and metabolism	140	34
Posttranslational modification, protein turnover, chaperones	242	32
Replication, recombination and repair	92	14
RNA processing and modification	0	0
Secondary metabolites biosynthesis, transport and catabolism	145	2
Signal transduction mechanisms	239	26
Transcription	354	36
Translation, ribosomal structure and biogenesis	103	12

Note: As a use case study, this analysis found that the VFs of *Brucella* (an intracellular bacterium that causes zoonotic brucellosis) are enriched in different metabolisms (e.g., amino acid transport and metabolism, carbohydrate transport and metabolism, inorganic ion transport and metabolism, etc.), suggesting the importance of these *Brucella* VFs overcoming the harsh intracellular metabolic environment in the host cells (1).

Supplemental Table 3. Predicted PPI interactions between human proteins and virulence factors in Victors.

Taxonomy	Species	Number of Virulence factors in Victor	Predicted interactions
**774**	*Bartonella bacilliformis*	1	0
**520**	*Bordetella pertussis*	2	0
**139**	*Borrelia burgdorferi*	4	0
**235**	*Brucella abortus*	27	254
**29459**	*Brucella melitensis*	115	1129
**29461**	*Brucella suis*	79	820
**13373**	*Burkholderia mallei*	2	0
**28450**	*Burkholderia pseudomallei*	3	0
**197**	*Campylobacter jejuni*	4	0
**5476**	*Candida albicans*	12	216
**1491**	*Clostridium botulinum*	2	470
**1717**	*Corynebacterium diphtheriae*	3	586
**1351**	*Enterococcus faecalis*	1	0
**562**	*Escherichia coli*	84	1412
**727**	*Haemophilus influenzae*	4	9
**210**	*Helicobacter pylori*	4	0
**446**	*Legionella pneumophila*	9	48
**1773**	*Mycobacterium tuberculosis*	11	192
**487**	*Neisseria meningitidis*	21	1507
**28901**	*Salmonella choleraesuis*	16	1765
**621**	*Shigella boydii*	15	511
**622**	*Shigella dysenteriae*	22	490
**623**	*Shigella flexneri*	30	523
**624**	*Shigella sonnei*	17	490
**1311**	*Streptococcus agalactiae*	12	1224
**1313**	*Streptococcus pneumoniae*	18	1549
**1314**	*Streptococcus pyogenes*	12	2144
**5811**	*Toxoplasma gondii*	2	0
**630**	*Yersinia enterocolitica*	3	0
**632**	*Yersinia pestis*	18	0

Supplemental Figures:

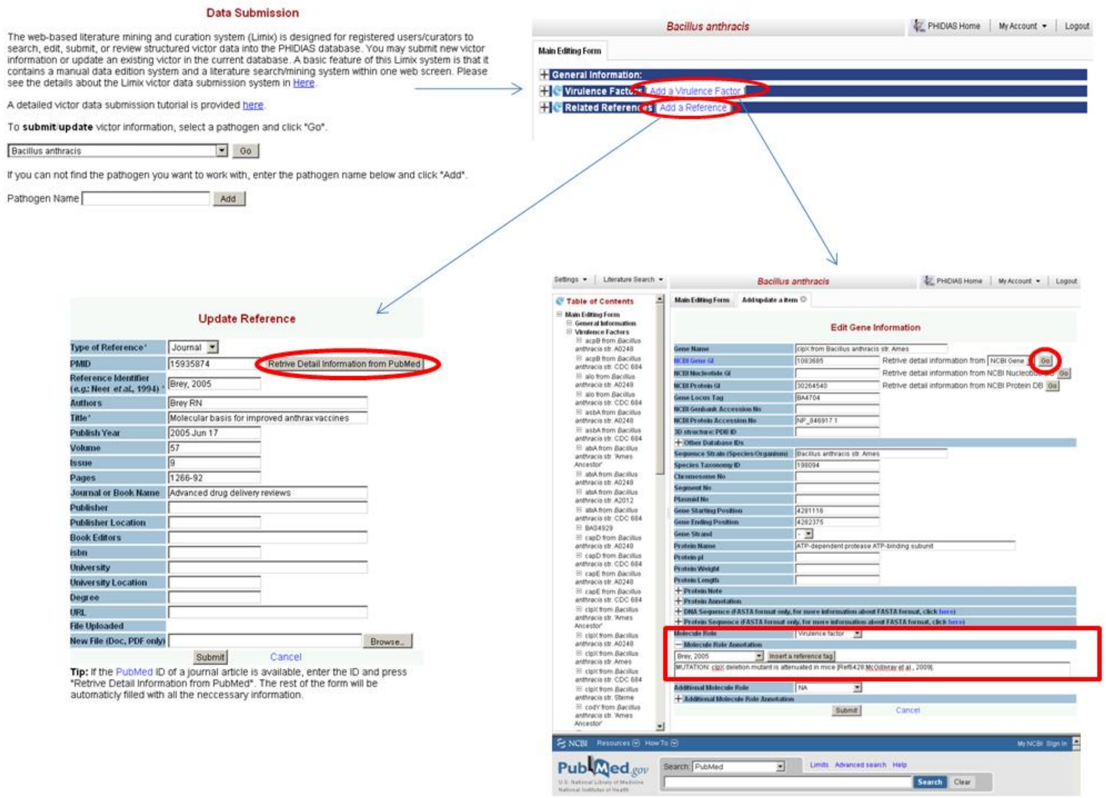

Supplemental Figure 1. Victors data curation, submission, and review processes. After a Victors curator logs into the Victors curation system, the curator can first select a pathogen and navigate to a web curation page where new virulence factors and references can be added. For the reference addition, a PubMed ID can be found and added. After clicking on "Retrieve detail information from PubMed", all citation information related to the PubMed ID will be automatically extracted from PubMed and added to the website form. Such a reference can then be added to the Virulence factor page using the "insert a reference tag" feature. Corresponding evidence information can then be annotated from the reference, edited, and submitted from the curation web page. Such information can only be available for the Public to review after expert review and approval through another data review webpage.

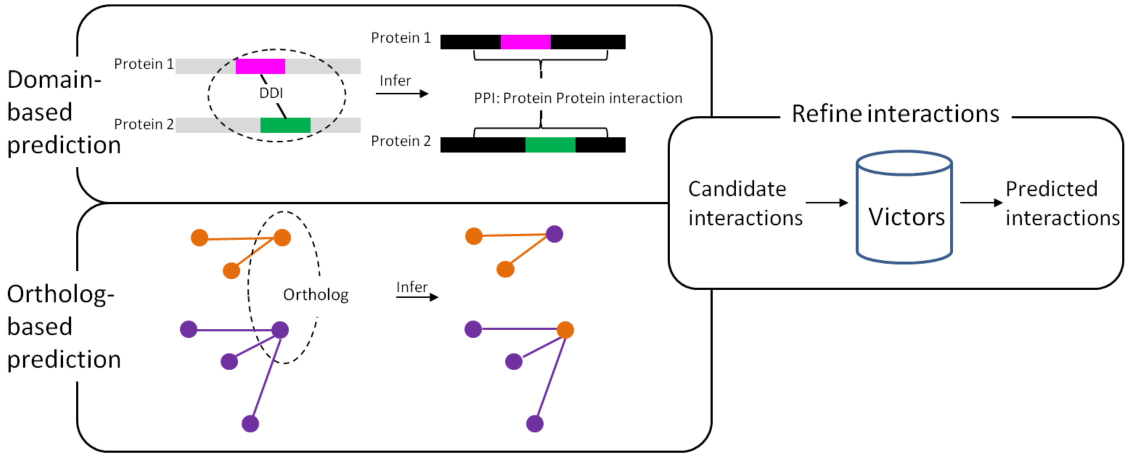

Supplemental Figure 2. Project workflow for predicting pathogen-human protein-protein interactions. For any two proteins, one from human and the other from a pathogen, it is inferred that these two proteins interact with each other when they contain interacted domains (domain-domain interaction or DDI), or if one protein is an ortholog of a third protein that interacts with the other protein. All proteins from 30 pathogens (Supplemental Table 2) were used to predict possible human-pathogen protein-protein interactions. The list of Victors virulence factors was used to filter out other pathogen proteins to refine the interaction results.

As domain is the conserved part of a protein, the "domain inferred" prediction is to infer inter-species PPIs from "domain-domain interactions" (DDI) (2). The computational algorithm works as follows:

① $A_{pathogen}$ protein has Domain Union U1{ $pu_1,pu_2,\ldots pu_n$ } and B_{human} protein has Domain Union U2{ $hu_1,hu_2,\ldots hu_n$ };
② If there are more than one DDI within U1 and U2: DDI(U1,U2) $\neq 0$ & >=1;
③ Then PPI($A_{pathogen}$, B_{human}) is true.

The "ortholog inferred" prediction is to infer the inter-species PPIs based on their orthologs interaction information. The orthologous pairs of proteins were defined by employing Inparanoid program (http://inparanoid.cgb.ki.se/) with default parameters (3). The PPI database HPRD (http://www.hprd.org/) was used to find the protein-protein interactions (4). The computational algorithm works as follows:

① OrthoU3{$ou_1,ou_2,\ldots ou_n$} is orthologous human proteins of $A_{pathogen}$ protein;
② If any member in OrthoU3 interacts with B_{human} protein: PPI(ou_x, B_{human});
③ Then PPI($A_{pathogen}$, B_{human}) is true.

Furthermore, both interactions generated by "domain inferred" and "ortholog inferred" methods were combined into a unique human-pathogen interactome.

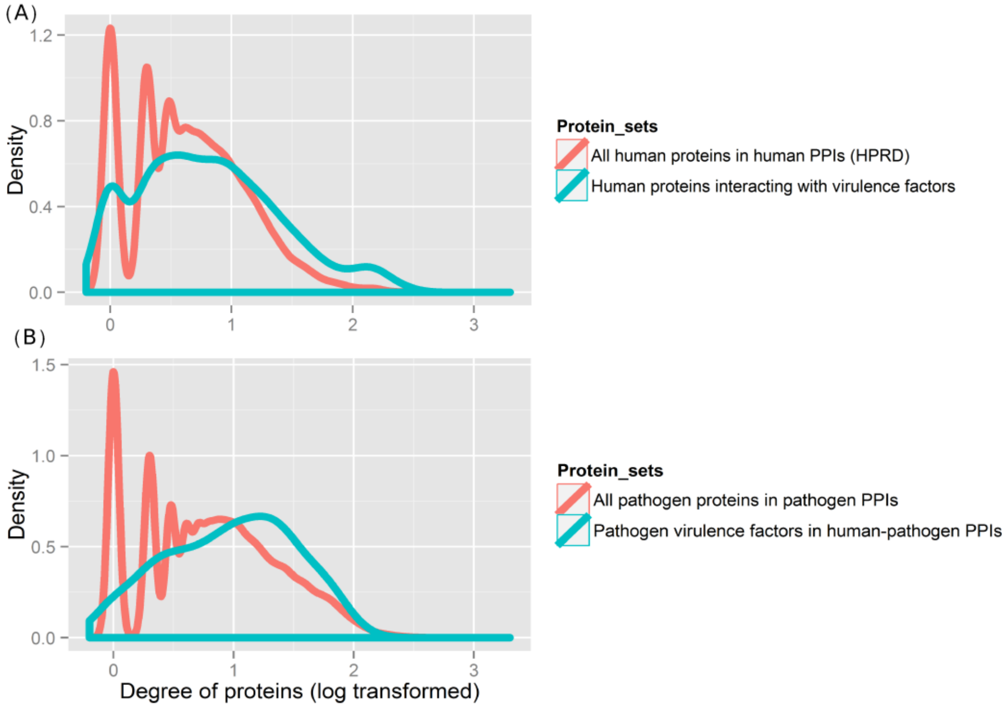

Supplemental Figure 3. The density curves of protein degrees. X-axis is the log10 transformed protein degrees (the degree of a node in a network is the number of connections it has); Y-axis is the density of protein. The density curve shows the probability of the degrees. For a specific value on the x-axis, the higher "Density" value on the y-axis, the higher probability it is. (A) Density curves of human proteins. Red line: All human proteins in human PPIs (PPIs in HPRD); Blue line: Human proteins interacting with virulence factors. (b) Density curves of pathogen proteins. Red line: All pathogen proteins in pathogen PPIs (using the pathogens in Supplemental Table 2); Blue line: Pathogen virulence factors in human-pathogen PPIs.

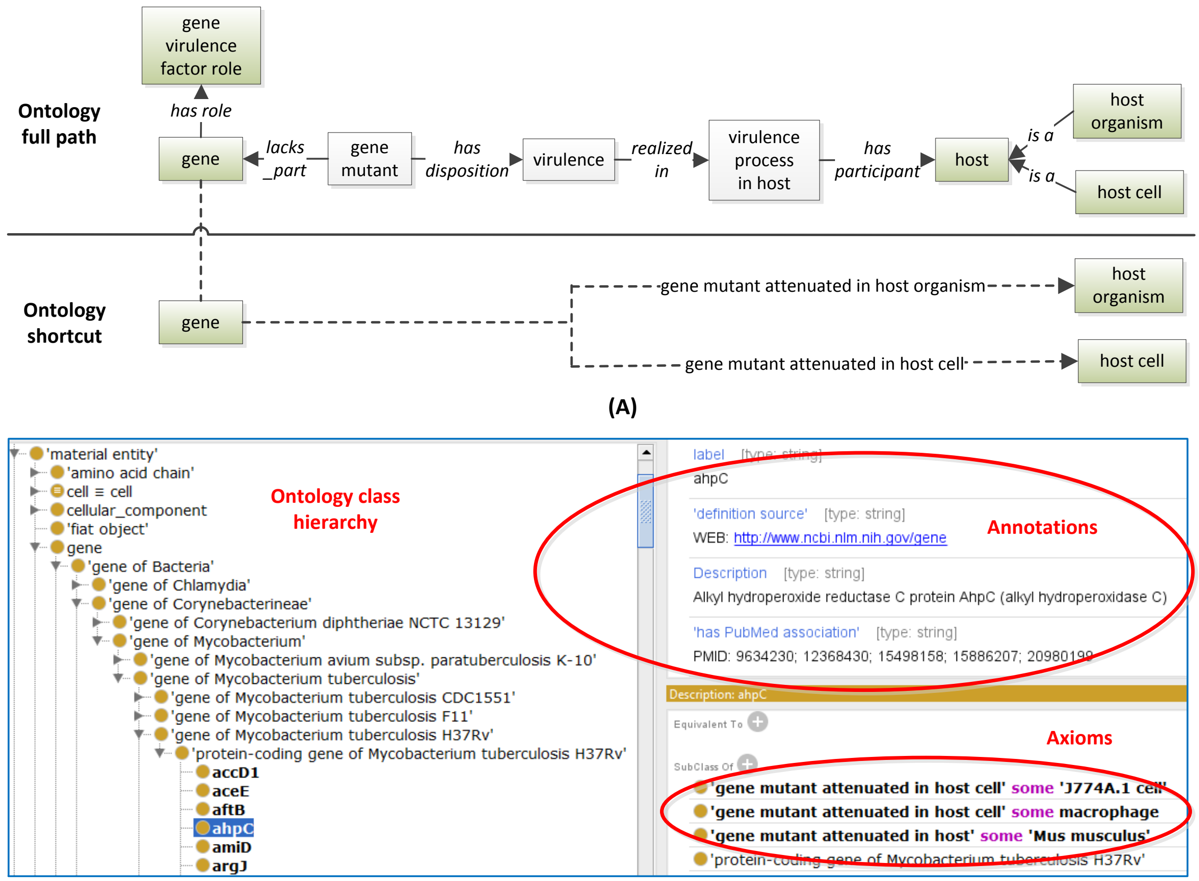

Supplemental Figure 4. OHPI ontology design pattern and illustration. (A) OHPI design pattern for representing VF and its interaction with host. (B) OHPI representation of *ahpC* gene, a virulence factor gene from *Mycobacterium tuberculosis* CDC1551. The section of annotations includes evidence, reference and other related information.

The Ontology of Host-Pathogen Interactions (OHPI) represents the virulence factors (VFs) collected in the Victors database and how these VFs interact with the host including host organism or host cell. This figure shows the general OHPI ontology design pattern which was then used to represent the information for individual VFs and their interactions with the host.

OHPI is developed by following the Open Biomedical Ontology (OBO) Foundry principles (e.g., openness and collaboration). OHPI reuses existing ontologies (5). OHPI uses the Basic Formal Ontology (BFO) (6) as the upper level ontology and aligns with the Infectious Disease Ontology (IDO) (7). OHPI also reuses terms from the Gene Ontology (GO) (8), Ontology of Genes and Genomes (OGG) (9), and Cell Line Ontology (CLO) (10) to represent cellular components, genes, and cell lines, respectively. OHPI includes object properties to semantically represent the relations between VFs and host entities (Supplemental Figure 4A). For example, the OHPI object property 'gene mutant attenuated in host cell' represents a relation between a gene and a host cell where the microbial mutant lacking the gene is attenuated in the host cell compared to the wild type microbe. Such an object property can be used to represent a VF and its interaction in a host cell, e.g., an oxidative stress response gene *ahpC* of *M. tuberculosis* strain H37Rv and mouse

macrophage cell line J774 cell line (Supplemental Figure 4B), where the *ahpC* mutant of strain H37Rv is attenuated in J774 cells (11).

The default OHPI project website is at GitHub: https://github.com/OHPI/ohpi/.
It is deposited at Ontobee: http://www.ontobee.org/ontology/OHPI, and BioPortal: http://bioportal.bioontology.org/ontologies/OHPI. As of August 15, 2018, OHPI contains 6704 terms, including 6562 classes, 87 object properties, 54 annotation properties. The detailed OHPI statistics can be found at the Ontobee page: http://www.ontobee.org/ontostat/OHPI.

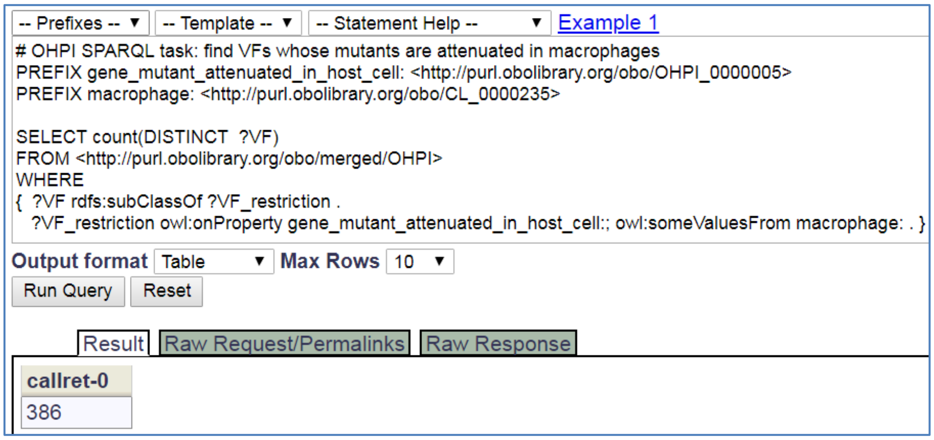

(A)

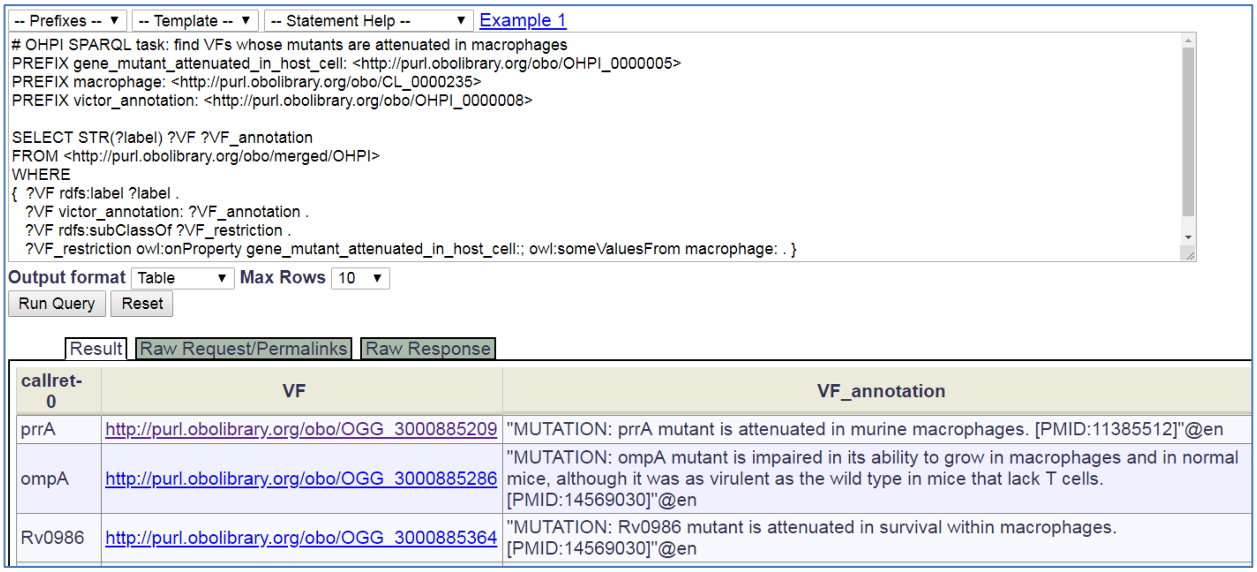

(B)

Supplemental Figure 5. OHPI SPARQL example. (A) Query the number of virulence factors whose mutants are attenuated in macrophages. (B) Query the gene label, gene ID in OGG, and VF annotations of those virulence factors whose mutants are attenuated in macrophages. The Cell Type (CL) ontology term CL_0000235 represents macrophages. The OHPI ontology term OHPI_0000005 represents the object property 'gene mutant attenuated in host cell'. The OHPI ontology term OHPI_0000008 represents the annotation property 'victor annotation'. This result shows that a script with a few lines of SPARQL code is able to query OHPI for useful information. The SPARQL query website is: http://www.phidias.us/ohpi/sparql/.

Using the machine-readable Web Ontology Language (OWL) format (http://www.w3.org/TR/owl2-quick-reference), OWL-based software programs can be developed to parse and extract the information from the ontology and offer advanced data analysis. For example, using an OHPI SPARQL program (http://www.phidias.us/ohpi/sparql/),

we could generate a simple SPARQL query script to identify 386 VFs whose mutants are attenuated in macrophages (Supplemental Figure 5A), and extract detailed information about the gene names, OGG IDs and VF annotations of these VFs (Supplemental Figure 5B).

References for Supplemental Materials:

1. He, Y. (2012) Analyses of Brucella pathogenesis, host immunity, and vaccine targets using systems biology and bioinformatics. *Frontiers in cellular and infection microbiology*, **2**, 2.
2. Zhao, X.M., Zhang, X.W., Tang, W.H. and Chen, L. (2009) FPPI: Fusarium graminearum protein-protein interaction database. *Journal of proteome research*, **8**, 4714-4721.
3. Ostlund, G., Schmitt, T., Forslund, K., Kostler, T., Messina, D.N., Roopra, S., Frings, O. and Sonnhammer, E.L. (2010) InParanoid 7: new algorithms and tools for eukaryotic orthology analysis. *Nucleic acids research*, **38**, D196-203.
4. Keshava Prasad, T.S., Goel, R., Kandasamy, K., Keerthikumar, S., Kumar, S., Mathivanan, S., Telikicherla, D., Raju, R., Shafreen, B., Venugopal, A. *et al.* (2009) Human Protein Reference Database--2009 update. *Nucleic acids research*, **37**, D767-772.
5. He, Y., Xiang, Z., Zheng, J., Lin, Y., Overton, J.A. and Ong, E. (2018) The eXtensible ontology development (XOD) principles and tool implementation to support ontology interoperability. *Journal of biomedical semantics*, **9**, 3.
6. Grenon, P. and Smith, B. (2004) SNAP and SPAN: Towards Dynamic Spatial Ontology. *Spatial Cognition and Computation*, **4**, 69-103.
7. Cowell, L.G. and Smith, B. (2010) In Sintchenko, V. (ed.), *Infectious Disease Informatics*. Springer, New York Dordrecht Heidelberg London, pp. 373-395.
8. Ashburner, M., Ball, C.A., Blake, J.A., Botstein, D., Butler, H., Cherry, J.M., Davis, A.P., Dolinski, K., Dwight, S.S., Eppig, J.T. *et al.* (2000) Gene ontology: tool for the unification of biology. The Gene Ontology Consortium. *Nature genetics*, **25**, 25-29.
9. He, Y., Liu, Y. and Zhao, B. (2014), *The 2014 International Conference on Biomedical Ontologies (ICBO 2014)*. CEUR Workshop Proceedings, Houston, TX, USA, Vol. 1327, pp. 13-20.
10. Sarntivijai, S., Lin, Y., Xiang, Z., Meehan, T.F., Diehl, A.D., Vempati, U.D., Schürer, T.C., Pang, C., Malone, J., Parkinson, H. *et al.* (2014) CLO: The Cell Line Ontology. *Journal of biomedical semantics*, **5**, 37.
11. Master, S.S., Springer, B., Sander, P., Boettger, E.C., Deretic, V. and Timmins, G.S. (2002) Oxidative stress response genes in Mycobacterium tuberculosis: role of ahpC in resistance to peroxynitrite and stage-specific survival in macrophages. *Microbiology*, **148**, 3139-3144.
